# Mixed-methods study exploring medium to longer-term outcomes following selective dorsal rhizotomy in ambulatory children with cerebral palsy at a tertiary hospital in the UK: MOSAiC study protocol

**DOI:** 10.1136/bmjopen-2025-108558

**Published:** 2025-12-08

**Authors:** Deepti Chugh, Cherry Kilbride, Hortensia Gimeno, Kristian Aquilina, Lucy Alderson, Tim Theologis, Eleanor Main

**Affiliations:** 1 Physiotherapy, Great Ormond Street Hospital for Children NHS Foundation Trust, London, UK; 2 Physiotherapy, III, University College London Great Ormond Street Institute of Child Health, London, UK; 3 Health Sciences, Brunel University London, Uxbridge, UK; 4 Queen Mary University of London, London, UK; 5 Neurosurgery, Great Ormond Street Hospital For Children NHS Foundation Trust, London, UK; 6 Physiotheray, ORCHID, Great Ormond Street Hospital for Children NHS Foundation Trust, London, UK; 7 Nuffield Department of Orthopaedics, Rheumatology and Musculoskeletal Sciences, University of Oxford, Oxford, UK

**Keywords:** Paediatric neurosurgery, Child, Patient Reported Outcome Measures, QUALITATIVE RESEARCH, REHABILITATION MEDICINE

## Abstract

**Abstract:**

**Introduction:**

Selective dorsal rhizotomy (SDR) is one of the treatment options available for spasticity management in ambulatory children and young people with cerebral palsy (CYPwCP). Although improvements in gross motor function one to two years after surgery have been established, evidence of longer-term benefit requires further investigation. Given the irreversible nature of SDR and the increased rehabilitation commitments required from families and clinicians, providing evidence of longer-term benefits is essential to support their decision-making. This study aims to investigate medium (3–5 years) and long-term (6–10 years) SDR outcomes in ambulatory children with CP and how SDR affects families’ lives over time.

**Methods and analysis:**

This is a convergent parallel mixed-methods study using the International Classification of Functioning, Disability and Health as a theoretical framework. The study aims to recruit 90 CYPwCP participants, who had SDR at a tertiary hospital in the UK when aged between 3 and 14 years. Participants (parents and CYPwCP) will be invited to complete an online survey and attend the hospital for one follow-up visit 3 or more years after SDR. Comparisons will be made with existing data on objective measures and parent-reported outcomes collected in clinical practice at baseline, 6, 12 and 24 months to understand the trajectory of changes. Semistructured interviews will be conducted with 18–20 parents/carers and 25–30 CYPwCP to understand their perspectives on the outcomes of SDR compared with their prior expectations. The Framework Method will be used to analyse qualitative data both inductively and deductively. Qualitative and quantitative study data will be integrated using joint displays.

**Ethics and dissemination:**

Ethical approval has been obtained through the Coventry and Warwick Research and Ethics Committee (24/WM/0078). Findings will be shared through international conferences, peer-reviewed journals, social media and dissemination events for families and CYP.

**Trial registration number:**

NCT06518889.

**STRENGTHS AND LIMITATIONS OF THIS STUDY**

- This mixed-methods study combines clinical outcomes with parent-reported and child-reported outcomes and experiences, prioritising the voices of children and young people with cerebral palsy.
- A multimodal data collection approach (online, paper, virtual and in-person) supports inclusive participation across diverse needs and contexts.
- Active involvement of a study advisory group of children, young people and parents with lived experience throughout design, analysis and dissemination enhances methodological rigour and relevance.
- The use of joint displays (convergence, divergence and silence) strengthens integration and interpretation of mixed-methods findings.
- Limitations include single-centre design, potential loss to follow-up due to contact changes and heterogeneity in time since selective dorsal rhizotomy and subsequent interventions, which may confound outcome attribution.

## Introduction

 Cerebral palsy (CP) is the most common physical disability with onset in childhood and has lifelong implications.^1^ It is a heterogeneous condition with varied clinical presentations that affect movement, posture and coordination, often leading to limitations in activity and participation.^2^ Spasticity is the most common neurological feature, affecting 70% of children and young people with CP (CYPwCP).^3^ Management typically focuses on reducing spasticity through a range of interventions, including oral medications, botulinum toxin A injections and neurosurgical interventions.^4^ Rehabilitation therapy remains the primary adjunct to optimise outcomes from these treatment approaches.^5^

Selective dorsal rhizotomy (SDR) is a neurosurgical procedure used to permanently reduce lower limb spasticity in CYPwCP. In the single-level approach, sensory nerve roots from L1 to S2 are identified and divided into rootlets. Approximately 60%–70% of sensory rootlets between L2 and S1 and 50% of the L1 sensory rootlets are selectively divided.^6^ SDR is typically offered to CYPwCP classified as Gross Motor Function Classification System (GMFCS) levels II and III,^7^ who are able to walk with or without walking aids. Surgery is followed by intensive rehabilitation for at least 2 years to optimise functional outcomes.^8^ The SDR procedure has been available at some centres around the world for over four decades, but was only commissioned by the National Health Service in England (NHSE) in 2018 for CYPwCP in GMFCS levels II and III, following a multicentre study.^9^ Under current NHSE guidelines, CYPwCP are only followed for 2 years after SDR and then discharged to their local community care without provisions for long-term monitoring. As these CYPwCP have not yet reached skeletal maturity, they remain at risk of developing deformities requiring further orthopaedic interventions.^10^ As such, the evidence on the longer-term outcomes of SDR within the UK healthcare context is lacking.

Short-term outcomes of SDR, up to 2–3 years after surgery, are well-established, particularly in reducing spasticity and improving gross motor function.^11 12^ However, evidence for longer-term benefits in gross motor function and participation remains inconclusive, as highlighted in a recent systematic review.^13^ Despite the lack of clear evidence for longer-term benefits of SDR over other interventions, SDR continues to be offered at various centres worldwide, supported by findings from long-term observational studies using a variety of outcome measures.^14 15^ In shared decision-making, clinical decisions regarding the suitability of the SDR procedure occur alongside families’ expectations. Parental decision-making is frequently influenced by hopes of long-term benefits such as reduced orthopaedic interventions and improved quality of life (QoL).^16^ This has also been a strong focus from our patient and public involvement (PPI) activities. Families regularly express the need for clearer information on longer-term outcomes to guide their decision-making.^16^

Long-term outcomes following SDR have been identified among the top 10 research priorities in both the Childhood Disability and Paediatric Lower Limb Surgery James Lind Alliance Priority Setting Partnerships.^17 18^ There is a knowledge gap in the evidence on the effects and impact of SDR on CYP’s physical functioning and QoL within the UK healthcare context in the medium to longer term. Under the current NHSE SDR pathway, CYPwCP are seen for up to 2 years at a specialist centre, after which care is managed by local community services.^9^ Currently, there is no national CP register in the UK to capture the longer-term outcomes of CYPwCP who have undergone SDR. Furthermore, CYPs and parents’ experiences, prior expectations and perceptions of outcomes of SDR and rehabilitation remain underexplored. Capturing the voices of CYPs and parents is central to strengthening the evidence base and better supporting their needs, and informing their decision-making and expectations.

### The MOSAiC study

This paper describes the protocol for a mixed-methods study in an established tertiary paediatric movement disorder service in London, UK. The aims of this study are (1) to investigate medium (3–5 years) to long-term (6–10 years) outcomes after SDR surgery in ambulatory children with CP and (2) to explore how SDR surgery affects families’ (CYPs and parents’) lives over time. The study will address three main research questions:

- What are the gross motor function, musculoskeletal and QoL outcomes 3–10 years after SDR in ambulatory CYPwCP?
- What are CYPs’ and parents’ experiences of SDR and rehabilitation 3–10 years after SDR in relation to their prior expectations and subsequent outcomes?
- How do gross motor function, orthopaedic and QoL outcomes in ambulatory CYPwCP compare with CYP and their parents’ perceptions, experiences and QoL 3–10 years after SDR surgery?

## Methods

### Study design and theoretical framework

**Figure 1:**
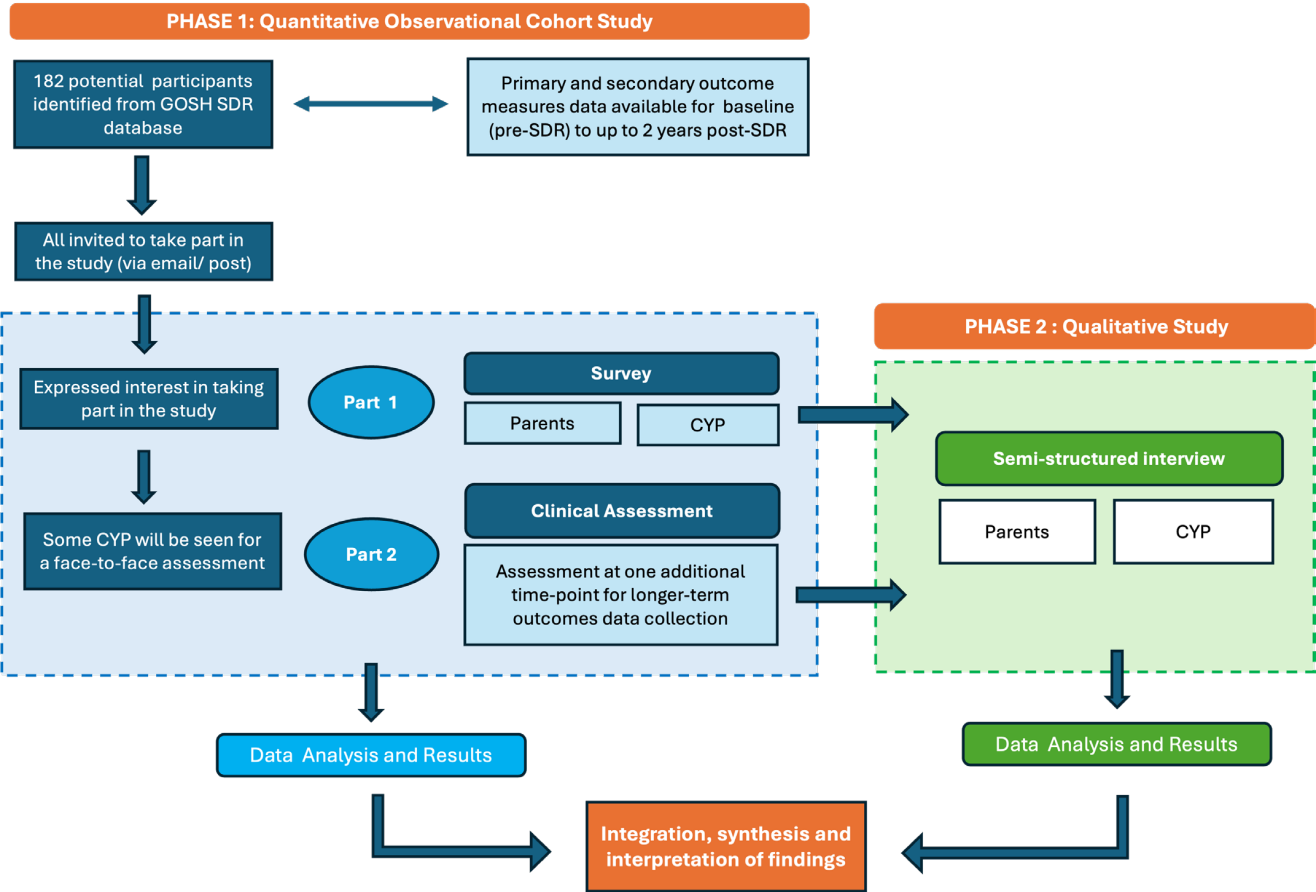
Mixed-methods study design. CYP, children and young people; GOSH, Great Ormond Street Hospital for Children; SDR, selective dorsal rhizotomy.

MOSAiC (**M**edium to longer term **O**utcomes following **S**elective dorsal rhizotomy in **A**mbulatory children w**i**th **C**erebral palsy) is an observational study using a mixed-methods convergent parallel study design, in which quantitative and qualitative data will be collected concurrently, analysed separately and then integrated and synthesised.^19^ The quantitative component (phase 1) comprises an observational cohort study, involving a survey and clinical assessments to objectively assess changes in gross motor function compared with baseline and previous assessments. The qualitative component (phase 2) involves semistructured interviews with CYPwCP and parents to explore their perspectives on the outcomes and lived experiences following SDR surgery (figure 1). The integration and synthesis of findings from both phases will bring new insights by leveraging the strengths of both methodologies.^20^ This dual approach is designed to generate a more comprehensive and holistic understanding of longer-term SDR outcomes, guided by the International Classification of Functioning, Disability and Health (ICF)^21^ as a theoretical framework. The ICF is a biopsychosocial model of functioning and disability and will underpin all aspects of this study, including study design, data collection, analysis and interpretation.

The study is philosophically grounded in pragmatism, which supports pluralistic approaches to knowledge generation by incorporating both subjective and objective perspectives.^22^ Consistent with this paradigm, equal priority will be given to both the quantitative and qualitative methodologies. The integration of these two components will occur at the data collection, results and analysis stages. A nested subsample from phase 1 will be purposefully selected for phase 2 interviews. The initial group-level and individual-level findings will further inform the development of the interview topic guide. Final interpretation will be derived through the synthesis of findings from both phases.

### Study sample and recruitment

This single-centre study will be conducted at Great Ormond Street Hospital for Children (GOSH), a paediatric tertiary hospital in London, UK. Inclusion criteria are CYPwCP who had SDR surgery at GOSH at least 3 years prior and were classified as GMFCS level II or III at the time of surgery. The GMFCS is a five-level ordinal classification system that categorises CYPwCP based on gross motor abilities, mobility limitations, need for assistive devices or wheeled mobility.^7^ Children in levels I and II are able to walk independently with some difficulty, while those at level III walk using a walking aid (online supplemental SI-1). The NHS England selection criteria for SDR eligibility are outlined in online supplemental SI-2. Participants will be eligible for this study if they completed a baseline assessment prior to SDR and have at least one follow-up assessment at 6, 12 or 24 months postsurgery. Parents of eligible CYPwCP will also be invited to participate. For those who have recently undergone orthopaedic surgery or received botulinum toxin injections in the lower limbs, data will be collected 3–9 months after that procedure, allowing time for recovery. This decision will be based on the type of procedure and in consultation with CYPwCP and their families, consistent with routine practice.

Based on the eligibility criteria, a minimum of 182 participants has been identified from the GOSH SDR database for inclusion in the observational cohort study (phase 1). All CYPwCP and parents participating in phase 1 will be eligible for the qualitative study (phase 2). Purposeful sampling will be used to identify CYPwCP and parents, ensuring a diverse representation of participants based on the GMFCS level, age at surgery, length of follow-up and outcomes at 2 years after SDR. The concept of ‘information power’, where the more relevant and high quality information the sample holds, the lower the number of participants needed, is used to determine the sample size.^23^ Based on this premise and previous qualitative research experience, a sample of approximately 18–20 parents and 25–30 CYPwCP is anticipated to provide maximum variation and depth to explore a range of experiences and perspectives.

### Recruitment process

All families attending the clinical service at GOSH who have previously agreed to be contacted for research will receive an invitation via email or post. A member of the clinical team will approach families and CYPwCP to confirm their interest in the study. If they agree to be contacted, the principal investigator (PI) (DC) will provide further study information. Families who agree to take part in the study will receive personalised survey links for both parents and CYP and will be invited to attend a face-to-face appointment at GOSH.

For the qualitative component (phase 2), a separate participant information sheet will be provided to eligible parents and CYP. Written informed consent will be obtained from parents, and consent or assent (as appropriate) from CYPwCP before data collection for both phases.

### Procedures

#### Phase 1: quantitative study

Participants (parents and CYPwCP) will complete an online survey via REDcap, followed by a single study visit for a clinical assessment. The clinical assessment will start with more physically demanding assessments, such as the 6-Minute Walk Test (6-MWT),^24^ Gross Motor Function Measure (GMFM),^25^ Timed Up and Go (TUG),^26^ followed by evaluation of muscle tone using the Modified Ashworth Scale (MAS)^27^ and lower limb passive joint range of movement. The assessment protocol mirrors standard clinical practice, and all participating CYPwCP will be familiar with this process. All assessments will be conducted by the PI (DC), supported by a clinical physiotherapist or physiotherapy assistant.

**Figure 2:**
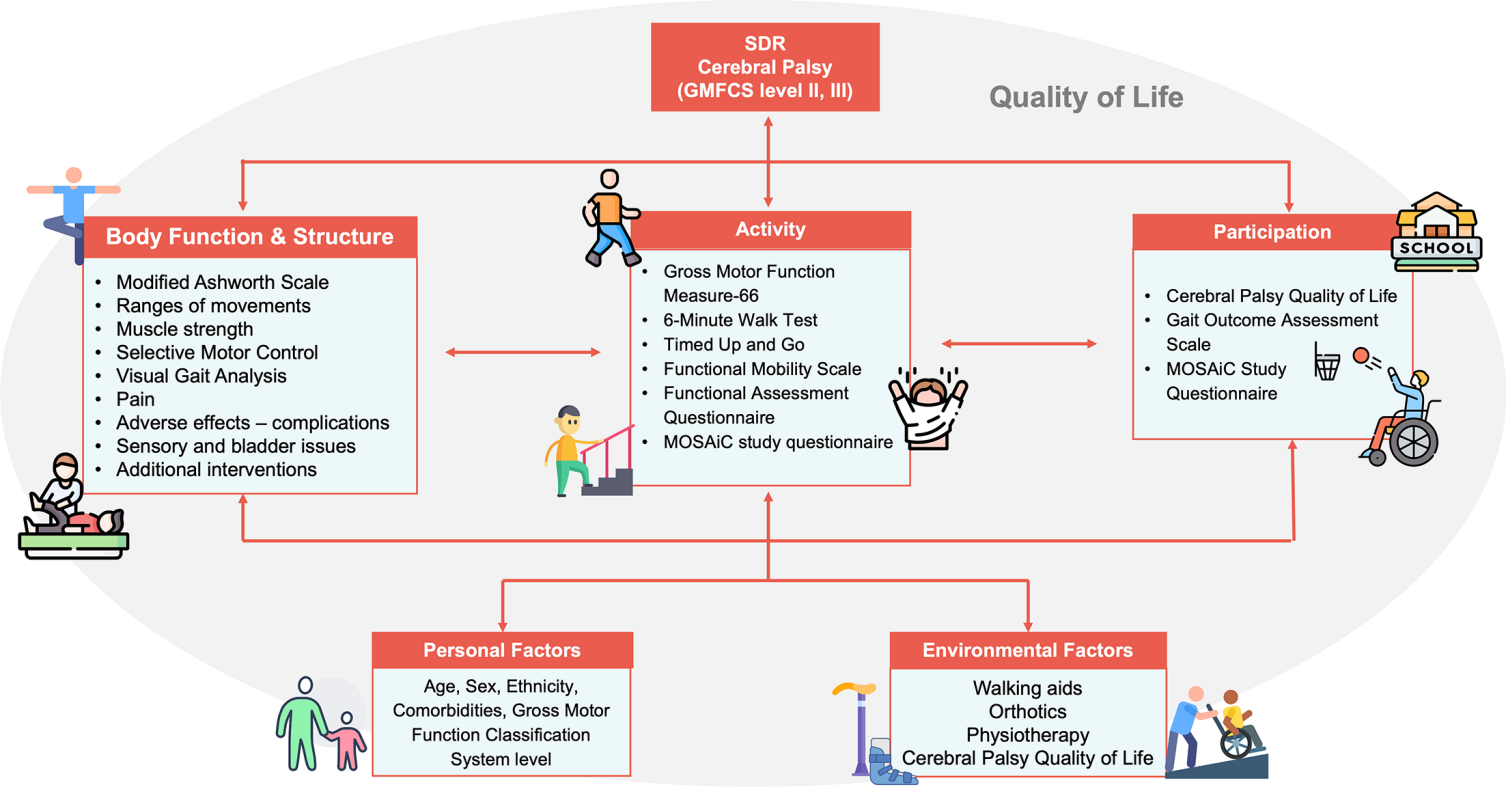
Distribution of outcomes across different domains of the ICF. GMFCS, Gross Motor Function Classification System; ICF, International Classification of Functioning, Disability and Health; SDR, selective dorsal rhizotomy; MOSAiC, Medium to longer term Outcomes following Selective dorsal rhizotomy in Ambulatory children with Cerebral palsy.

The outcomes and outcome measures were selected based on the findings from a scoping review on the outcomes used in the SDR literature,^28^ NHSE commissioning guidelines and acceptability and meaningfulness to parents and CYPwCP (as informed by the PPI and study advisory group). The selected outcomes and measures are presented in table 1 and the mapping of outcomes across the domains of the ICF is illustrated in figure 2.

**Table 1:** Outcomes measures included in this study

	Measure	Administration
Classification	Gross Motor Function Classification System–Family report Questionnaire	Questionnaire
Primary outcome measures	Gross Motor Function Measure-66 and centiles	Clinician-assessed
	6-Minute Walk Test	Clinician-assessed
	Cerebral Palsy Quality of Life	Questionnaire–Parent-proxy and CYP-reported
	MOSAiC Study Questionnaire	Questionnaire–Parent-reported
Secondary outcome measures	Modified Ashworth Score	Physical examination
	Timed Up and Go	Clinician-assessed
	Edinburgh Visual Gait Analysis	Clinician-assessed
	Gait Outcome Assessment List–Child Version	Questionnaire–CYP-reported, with assistance from parent carer, if needed
	Functional Mobility Scale	Questionnaire–Parent and CYP-reported
	Functional Assessment Questionnaire	Questionnaire–Parent-reported

CYP, children and young people; MOSAiC, Medium to longer term Outcomes following Selective dorsal rhizotomy in Ambulatory children with Cerebral palsy.

A study-specific questionnaire (online supplemental SI-3) has been developed in collaboration with the study advisory group of CYP and parents. The questionnaire is designed to capture a comprehensive range of post-SDR outcomes across all ICF domains. The survey content was shaped by the PPI and stakeholder activities, literature review and clinical experience. It includes items related to orthopaedic and pharmacological interventions required after SDR, bladder and bowel function, and sensory issues. The questionnaire also captures current orthotics and mobility aids, physiotherapy input and participation in physical leisure activities. Based on the recommendations of the advisory group members, questions were added to explore the psychological impact of SDR on CYP and their parents, as well as the impact on activities of daily living and engagement in education settings.

Parents and CYPwCP can complete the survey at any point, before, during or after their hospital visit. To accommodate family preferences and reduce any language or accessibility barriers, the questionnaire may be completed on paper, via phone, or in a virtual session with the PI (DC) and a translator. The survey will take approximately 30–50 min to complete. A thank-you voucher will be given to families as a token of appreciation for their time.

#### Primary outcome measures

GMFM-66: The GMFM-66 is a standardised clinical and research tool used to evaluate changes in gross motor function in CYPwCP.^25^ It consists of 66 items assessing a range of gross motor abilities, from lying, rolling, sitting and crawling to walking and jumping. Each item is scored using a 4-point criterion-referenced scoring system on a 0–3 ordinal scale. Final scores will be calculated using the GMFM Scoring App, which converts individual item scores into a total score ranging from 0 to 100, representing low to high gross motor ability, with interval-level measurement properties.^29^ The GMFM-66 is shown to be highly reliable, with intraclass correlation coefficients (ICCs) of 0.99 for test–retest reliability.^29^ The minimum clinically important difference (MCID) for GMFM-66 is estimated to be 1.5 and 1.2 units, corresponding to large effect sizes, for GMFCS level II and III, respectively.^30^

GMFM centiles: The GMFM centile curves represent the relationship between age and gross motor function, as measured by GMFM-66 scores, across different GMFCS levels. These curves depict the average trajectory of motor development, including both the rate of improvement and the maximum expected functional capacity, for CYPwCP between the ages of 2 and 12 years. For those classified as GMFCS level II, GMFM-66 scores typically plateau by around 9 years of age, whereas children in GMFCS level III typically reach peak scores by 8 years of age, followed by a potential decline of approximately 4.7 units during adolescence and into adulthood.^31^ Given the absence of reference data beyond age 12, centiles for 12-year-olds are often applied to older children in research.^32 33^

6-MWT: The 6-MWT is a self-paced, submaximal exercise test used to measure functional walking capacity and endurance in CYPwCP.^24 34^ The test will be conducted on a 25 m track at GOSH, using standardised instructions. Typical walking aids and orthoses, if applicable, will be used, and the distance walked in 6 min will be recorded. Reference values for children aged 4–17 years^35^ and longitudinal developmental trajectories and age-specific reference percentiles for CYPwCP across different GMFCS levels I–III have been published.^36^ The 6-MWT has excellent test–retest reliability (ICC=0.98).^24 34^ The MCID for the 6MWT in CYPwCP is not well established; a range of 20–36 m for GMFCS level II and 23–46 m for level III has been reported in the literature.^37^

Cerebral Palsy Quality of Life (CPQOL): The CPQOL is a validated tool designed to assess health-related QoL for CYPwCP across 5–7 domains.^38^ These include social well-being and acceptance, feelings about functioning, participation and physical health, emotional well-being and self-esteem, access to services, and pain and impact of disability. Items are rated on a nine-point rating scale, with domain scores calculated by averaging responses and converting them to a 0–100 scale, where higher scores indicate better QoL. There are two self-report versions, CPQOL-Child (for children aged 9–12 years) and CPQOL-Teen (for adolescents aged 13–18 years), and two proxy-report versions, completed by primary caregivers for children aged 4–12 and adolescents aged 13–18. The CPQOL has good internal consistency, ICC ranging from 0.74 to 0.92 for children and 0.81 to 0.96 for teens, and adequate test–retest reliability ranging from 0.76 to 0.89 for children and 0.59 to 0.83 for teens.^38 39^ Currently, there are no published MCID values for the CPQOL.

#### Secondary Outcome Measures

MAS: The MAS is a widely used clinical tool for assessing muscle tone by measuring resistance to passive movement during a high-velocity stretch.^27^ The limb is moved through its available range of motion, and resistance is graded on a 6-point ordinal scale, from 0 (no increase in tone) to 4 (rigid limb). The scale includes an intermediate score of 1+to capture subtle increases in tone. The inter-rater agreement has been reported with a mean ICC of 0.686 (95% CI 0.563 to 0.780) and a kappa coefficient (κ) of 0.360 (95% CI 0.241 to 0.468). The intrarater agreement shows an ICC of 0.644 (95% CI 0.543 to 0.726) and a κ of 0.488 (95% CI 0.370 to 0.591).^40^ The MCID for MAS in CYPwCP has not been established.

TUG: The TUG is a functional mobility test used to evaluate dynamic balance and anticipatory postural adjustments in CYPwCP.^26^ Participants are instructed to rise from a chair, walk 3 m to touch the wall and return to sit down as quickly as possible, without running. Usual walking aids and orthoses are used as needed. The TUG has excellent inter-rater reliability (ICC: 0.83–0.99).^41^ Minimal detectable change values range from 1.40 to 8.74 s, and MCID estimates range from 0.22 to 5.31 s in children aged 3 to 10 years.^41^

Edinburgh Visual Gait Analysis (EVGS): The EVGS is a video-based observational tool used to assess gait deviations in CYPwCP.^42^ It consists of 17 gait parameters measured at six anatomic levels (foot, ankle, knee, hip, pelvis and trunk) representing key features of pathological gait in CYPwCP. Each parameter is scored on a 3-point ordinal scale (0=normal, 1=moderate deviation, 2=marked deviation), resulting in a total score ranging from 0 (normal gait) to 34 (maximal deviation). The EVGS has excellent inter-rater (ICC2,1=0.90–0.97) and intrarater (ICC2,1=0.91) reliability.^43^ There is a large variation in the MCID values reported in the literature, ranging from 2.4 to 15 points. The MDC^90^ of 6.0^43^ and the MCID of 2.4 points^44^ have been proposed.

Gait Outcome Assessment List (GOAL) (child version): The GOAL is a validated questionnaire designed to determine gait-related priorities and functional mobility for ambulant CYPwCP.^45^ It comprises 48 items across seven domains, with scores standardised on a 0–100 scale, where higher scores indicate better function. Item scores are summed and averaged to generate domain-specific and total scores. Moderate to strong test–retest reliability has been reported for the parent version (ICC, total score: 0.98 and domain scores: 0.69–0.93).^45 46^

Functional Mobility Scale (FMS): The FMS is a validated tool used to assess functional mobility in CYPwCP, aged 4–18 years, across three distances: 5 m, 50 m and 500 m, representing typical home, school and community distances. For each distance, the use of assistive devices is rated on a 6-point ordinal scale, where 1 indicates wheelchair use and 6 indicates independent ambulation.^47^ The FMS demonstrates strong inter-rater reliability with quadratic weighted kappa coefficients ranging from 0.86 to 0.92 for the three distances.^48^ A change of one level is considered a clinically meaningful change.^49^

Functional Assessment Questionnaire (FAQ): The FAQ is a 10-point ordinal scale used to rate a child’s typical walking ability in community environment.^50^ It is a performance-based measure, with scores ranging from 1 (cannot take any steps at all) to 10 (walks, runs and climbs on level and uneven terrain without difficulty or assistance). It exhibits good inter-rater reliability (ICC=0.81) and intrarater reliability (ICC=0.92).^50^

#### Phase 2: qualitative data collection

Semistructured interviews will be conducted with a subset of parents and CYP using a topic guide (online supplemental SI-4), developed in collaboration with the study advisory group. Participants may be recruited from the same or different families. Parent interviews will explore their prior expectations of SDR and reflections, experiences and perceptions of the SDR surgery, post-SDR rehabilitation and any subsequent interventions. The interviews will also delve into perceived facilitators, barriers and challenges encountered during the SDR rehabilitation journey. Parents’ preoperative goals and expectations, satisfaction with outcomes and perspectives on their QoL will be explored.

Interviews with CYP will be adapted for their age, cognitive levels and communication abilities. Semistructured interviews will be used to discuss their participation in daily life, feelings about their abilities and challenges, social interaction and future goals. CYP will be offered the choice of having their main carer present in a supporting capacity. While older CYP are likely to engage in conversations, younger children will be offered the use of creative adjuncts such as drawing, ‘Play-Doh’, Lego, personal photographs and videos taken by the family as a memory aid. These interviews will prioritise the voice of CYP.

The interviews will be conducted either in person or virtually, according to the preferences of parents and CYP. Interviews will last approximately 30–60 min for CYP and 45–90 min for parents. All interviews will be audio-recorded and transcribed verbatim by an external transcription service approved by the Trust’s Research and Development department. Transcripts will be checked for accuracy against the recordings by the PI (DC). The researcher will also take notes during the interview and write a postinterview reflection to support the qualitative analysis.

### Data analysis

The data from the quantitative and qualitative phases will be analysed separately before being integrated during the interpretation phase, in line with the convergent parallel mixed-methods research design.

#### Phase 1: quantitative data analysis

Descriptive analysis will be used to provide participants’ demographic and baseline characteristics. Continuous variables will be summarised using mean and SD when normally distributed, or median and IQR when skewed. Categorical variables will be presented as frequencies and percentages. Association between categorical variables will be examined using cross-tabulation and χ² tests. The primary and secondary outcome measures (GMFM-66, GMFM-66 centile scores, 6-MWT, CP QOL, TUG, FMS and FAQ) will be compared between baseline and subsequent follow-up time points. Generalised linear equation models will be used to analyse longitudinal data across multiple time points. Univariate analysis will be conducted to explore associations between individual predictors and outcome variables. Non-parametric tests will be applied to ordinal outcome measures (FMS, FAQ and SMC). A multiple regression model will be created to identify predictors of favourable outcomes, incorporating relevant covariates.

#### Phase 2: qualitative data analysis

The framework method^51^ will be used for analysis. This method follows a systematic data analysis process and allows both inductive and deductive approaches. It will enable the integration of additional contextual data, such as field notes, subjective comments and reflections provided by families during face-to-face assessments. The framework method analysis will be used in conjunction with the theoretical framework (ICF) to inform theme development and interpretation. The analytical process will follow the established seven steps: (1) transcription, (2) familiarisation with the interview, (3) coding, (4) developing a working analytical framework (based on the interview topic guide and the theoretical framework-ICF), (5) applying the analytical framework, (6) charting data into the framework matrix where each column is a theme and each row is a participant’s transcript and (7) interpreting the data. The coding will be completed manually and managed using NVivo software. To ensure rigour and credibility, a subset of anonymised transcripts will be reviewed for coding and theme development by other members of the research team (CK, HG and EM), supporting reflexivity and consistency in interpretation.

#### Integration of findings

**Figure 3:**
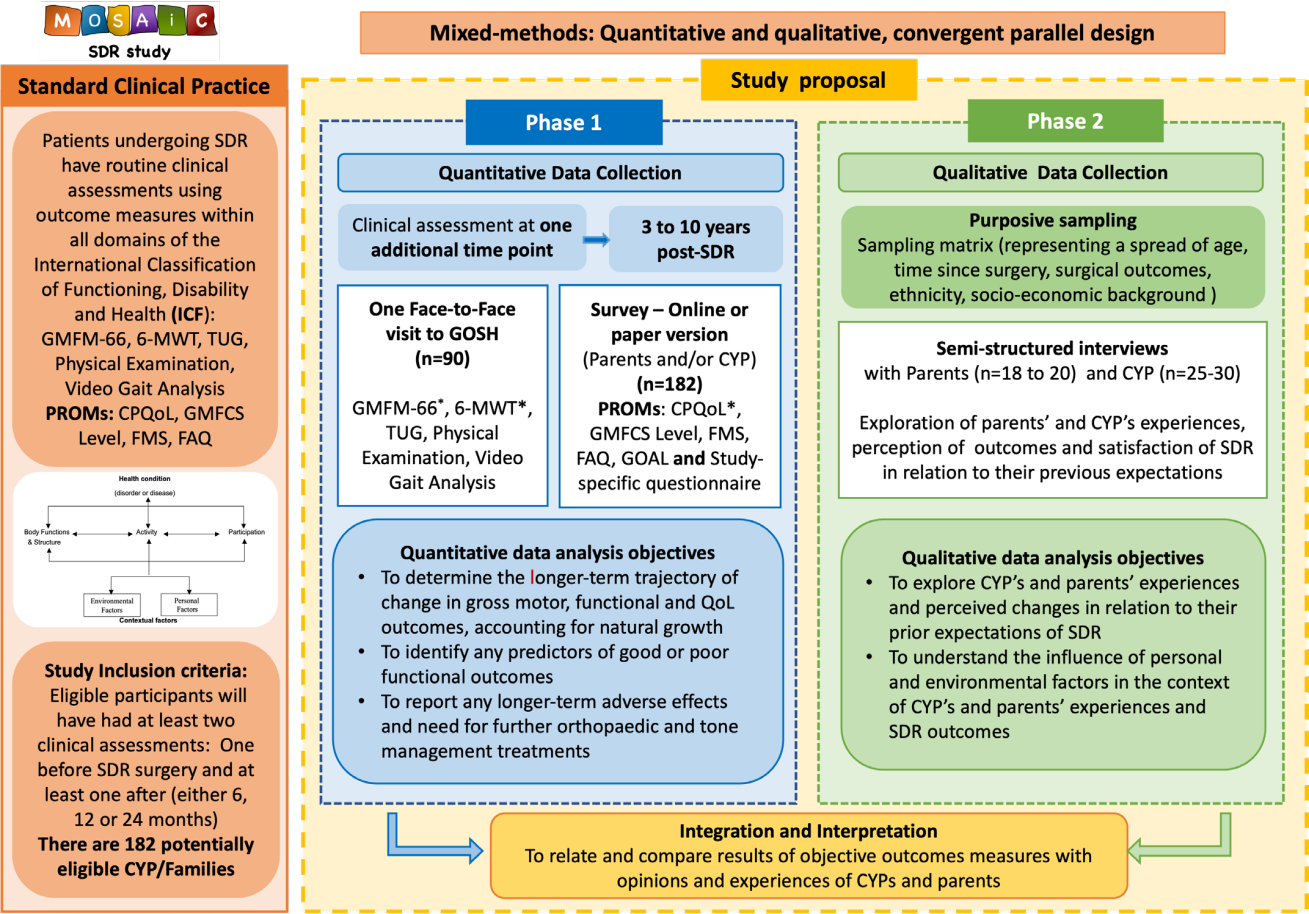
MOSAiC Study Summary diagram. 6-MWT, 6-Minute Walk Test; CPQOL, Cerebral Palsy Quality of Life; CYP, children and young people; FAQ, Functional Assessment Questionnaire; FMS, Functional Mobility Scale; GMFCS, Gross Motor Function Classification System; GMFM-66, Gross Motor Function Measure-66; GOAL, Gait Outcome Assessment List; GOSH, Great Ormond Street Hospital for Children; PROMs, Participant-Reported Outcome Measures; QOL, quality of life; SDR, selective dorsal rhizotomy; TUG, Timed Up and Go. *Primary outcome measures.

Findings from the quantitative and qualitative phases will be integrated through side-by-side comparisons using a summary table. The PI (DC) will systematically identify areas of convergence (agreement across datasets), divergence (contradictions) and silence (themes or findings present in only one dataset).^52^ The triangulation of quantitative and qualitative methods will lead to greater depth and credibility.^53^ Joint displays, a visual means of integrating data and representing mixed-methods results, will be used to generate new insights beyond the information gained from separate quantitative and qualitative results.^54^ This will provide rich insight into understanding the impact of SDR in the daily lives of CYPwCP and their parents. These new insights will be discussed within the research team and with the members of the study advisory group to ensure alignment with lived experiences, to enhance trustworthiness and credibility. Conclusions will be developed through meta-inferences, where interpretations are grounded in the meaning of combined findings.^55^ Good reporting of a mixed-methods study will be used to report findings to ensure methodological transparency.^56^ An overall summary of the research study protocol is presented in figure 3.

### Patient and public involvement

PPI has been integral to identifying and prioritising the research question and study design. PPI members include three CYPwCPs and three parents of CYPwCPs who had SDR surgery. In addition to this core advisory group, other parents and CYP have contributed at various stages, including reviewing patient-facing materials, providing feedback on the study questionnaires and informing the development of the interview topic guide. The study advisory group also confirmed that the research burden, time taken to complete the questionnaires, a single hospital visit and optional participation in interviews are reasonable and feasible from the families’ perspective. The study advisory group will remain involved throughout the project, advising on recruitment strategies, reviewing and commenting on emerging findings, supporting dissemination of findings and coproducing information resources to support other families.

## Ethics and dissemination

This study has received ethical approval (IRAS: 331711/ REC: 24/WM/0078) and is registered on ClinicalTrials.gov (NCT06518889). All parent and CYPwCP reported outcomes and study questionnaires are uploaded onto REDCap at GOSH. Study findings will be presented at national and international conferences and published in peer-reviewed, open-access journals. The study patient advisory group will play a key role in sharing results with wider CP and SDR communities via social media, patient forums and local and national SDR family days.

## Discussion

This paper outlines the research protocol for the MOSAiC study, a mixed-methods study involving CYPwCP who have undergone the SDR procedure at a tertiary children’s hospital in the UK. Outcomes across all domains of the ICF and QoL will be measured to understand the longer-term impact of SDR on CYPwCP and their families’ lives. The mixed-methods design enables the exploration of both measurable outcomes and personal experiences with a specific emphasis on CYPwCP’s voices, which are underrepresented in the SDR research.

This study will address an important research question identified during consultations with families. The evidence-based knowledge generated will directly inform clinical practice and become part of the conversations between healthcare professionals and families. It will support shared decision-making regarding the suitability of SDR for each child and prepare families for potential short-term and long-term outcomes, including the need for further pharmacological or orthopaedic interventions. A better understanding of the individual CYPwCP’s variability and the factors influencing outcomes will empower clinicians, CYP and their families to set achievable and realistic goals. These insights will improve patient selection for SDR, set realistic expectations and lead to better experiences for CYP and their families. Additionally, CYPwCP and family narratives will highlight any disparities in healthcare access and service delivery, identify inequities in the national health systems and help guide future resource allocation.

A key strength of this study lies in its mixed-methods approach. The integration of quantitative findings with qualitative insights will lead to a more complete and nuanced understanding of the impact of SDR than using one method alone. Inclusion of CYPwCPs and parents’ perspectives enhances the relevance and authenticity of findings. The outcome measures used in this study are consistent with those used at other centres in the UK and internationally, allowing for comparison of outcomes with other centres and future collaborations.

The absence of a comparison group from another site may limit the generalisability of the findings. However, since all centres in the UK follow similar SDR surgical and rehabilitation protocols, the sample of this study will be compared with cohorts from other centres.^12^ Potential sampling bias will be addressed through sensitivity analysis, by comparing baseline and 2-year post-SDR clinical outcomes of CYPwCP who do not participate in this study.

We anticipate that the MOSAiC SDR study will generate new and clinically relevant knowledge to support clinicians and families. The findings will help improve patient selection for SDR, set realistic expectations and lead to better experiences for CYP and their families.

### Status of study

Recruitment and data collection for phase 1, quantitative study began in June 2024 and will end in October 2025. Phase 2, involving semistructured interviews, runs from July to November 2025. Data analysis will start in November 2025.

## Supplementary material

10.1136/bmjopen-2025-108558 online supplemental file 1

10.1136/bmjopen-2025-108558 online supplemental file 2

10.1136/bmjopen-2025-108558 online supplemental file 3

10.1136/bmjopen-2025-108558 online supplemental file 4
