# Supplementary material for: Mixed-methods study exploring medium to longer-term outcomes following selective dorsal rhizotomy in ambulatory children with cerebral palsy at a tertiary hospital in the UK: MOSAiC study protocol
Source: BMJ Open. 2025 Dec 8;15(12):e108558. doi: 10.1136/bmjopen-2025-108558 (PMC12699567; doi:10.1136/bmjopen-2025-108558)

**Supplementary Information- 1**

https://canchild.ca/wp-content/uploads/2025/03/GMFCS_English_Illustrations_V2_-May1-2023-ACCESS.pdf


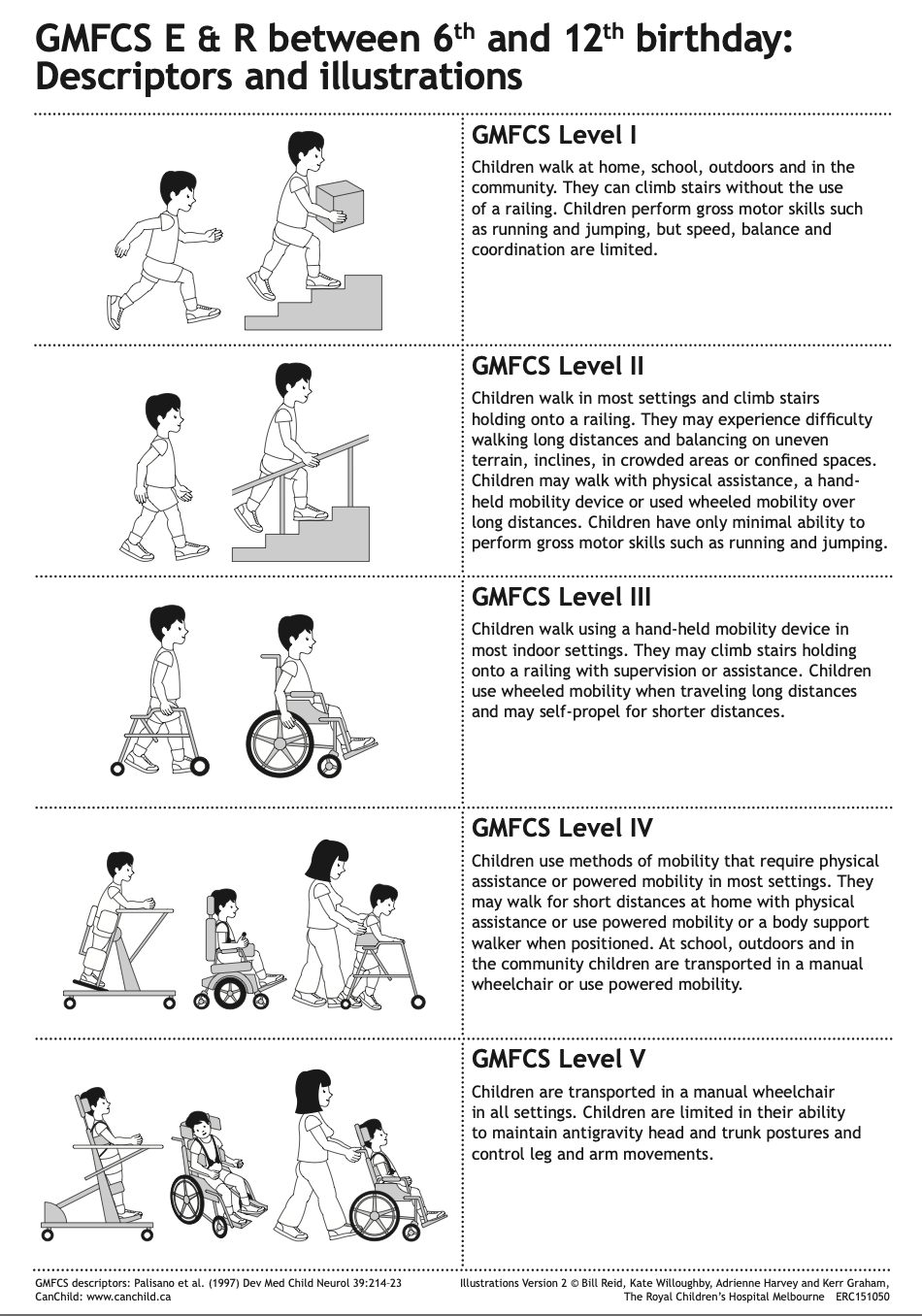


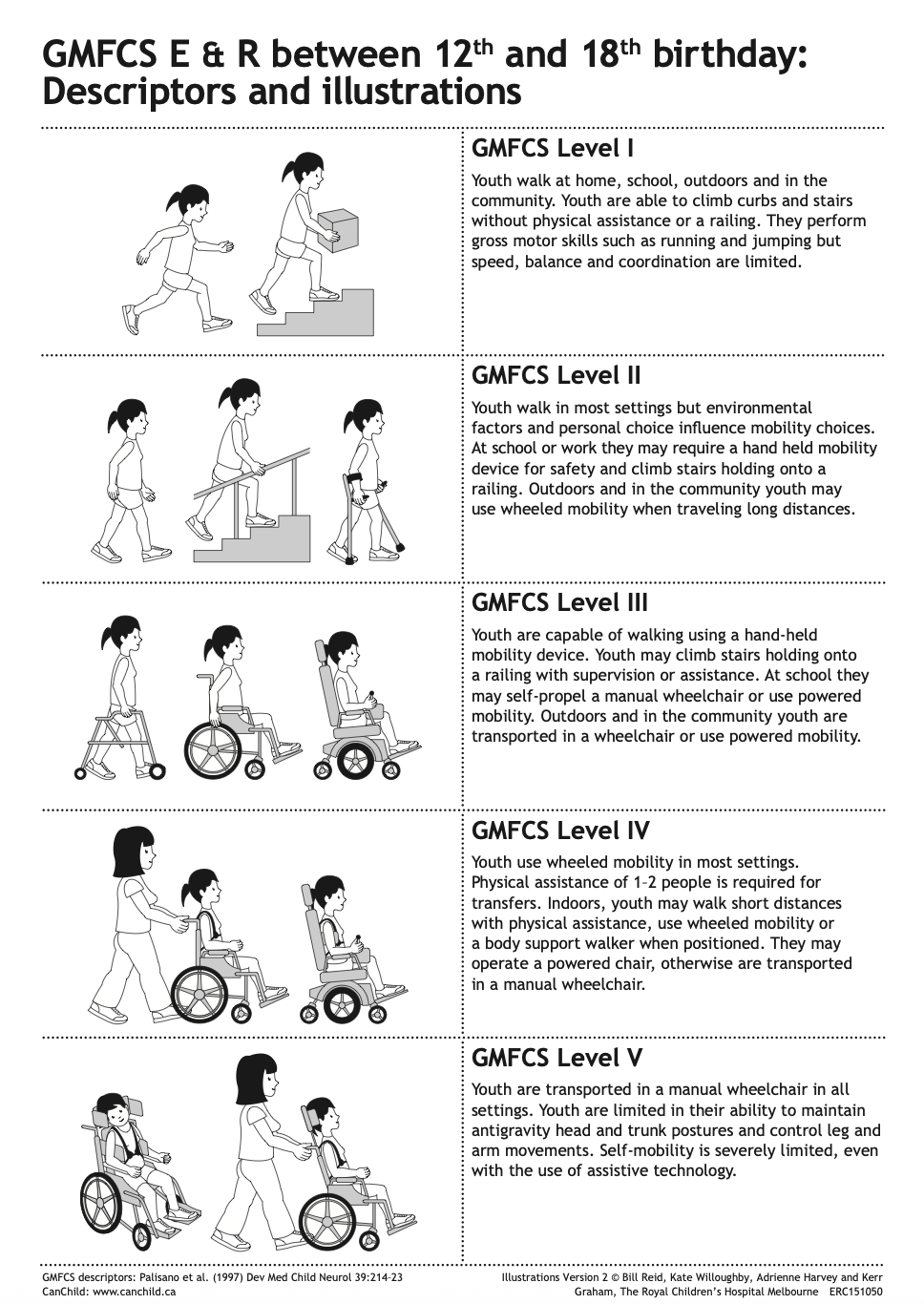

Supplement: online supplemental file 1 [file bmjopen-15-12-s001.docx]
