# Supplementary material for: Mixed-methods study exploring medium to longer-term outcomes following selective dorsal rhizotomy in ambulatory children with cerebral palsy at a tertiary hospital in the UK: MOSAiC study protocol
Source: BMJ Open. 2025 Dec 8;15(12):e108558. doi: 10.1136/bmjopen-2025-108558 (PMC12699567; doi:10.1136/bmjopen-2025-108558)
Supplement: online supplemental file 2 [file bmjopen-15-12-s002.docx]

**Supplementary Information- 2**

NHS England Selection criteria for SDR:

( https://www.england.nhs.uk/wp-content/uploads/2019/03/Selective-dorsal-rhizotomy-for-the-treatment-of-spasticity-in-cerebral-palsy-children-aged-3-9-years.pdf)

SDR is routinely commissioned for the treatment of spasticity in cerebral palsy,

mainly affecting the legs, in children functioning at GMFCS levels II and III.

Patients must meet the following criteria:

a) The child is aged 3 years to 9 years inclusive with a diagnosis of cerebral palsy

with spasticity mainly affecting the legs

b) The child has dynamic spasticity in lower limbs affecting function and mobility

and no dystonia

c) The MRI brain scan shows typical cerebral palsy changes and no damage to key

areas of brain controlling posture and coordination *

d) The child functions at GMFCS level II or III

e) There is no evidence of genetic or neurological progressive illness

f) The child has mild to moderate lower limb weakness with ability to maintain

antigravity postures;g) The child has no significant scoliosis or hip dislocation (Reimer’s index should be <40%)

* The typical MRI changes are those of white-matter damage of prematurity or

periventricular leucomalacia (PVL).

* Lesions in basal ganglia or cerebellum are contra-indications to SDR, since

they are associated with other cerebral palsy types (dystonia / ataxia).
