# Supplementary material for: Mixed-methods study exploring medium to longer-term outcomes following selective dorsal rhizotomy in ambulatory children with cerebral palsy at a tertiary hospital in the UK: MOSAiC study protocol
Source: BMJ Open. 2025 Dec 8;15(12):e108558. doi: 10.1136/bmjopen-2025-108558 (PMC12699567; doi:10.1136/bmjopen-2025-108558)
Supplement: online supplemental file 3 [file bmjopen-15-12-s003.pdf]

Supplementary Information -3

MOSAiC SDR Study Questionnaire

SDR study

Medium to long term Outcomes of Selective dorsal rhizotomy in Ambulatory children and young people with Cerebral palsy: A mixed-methods study

This questionnaire is designed to help us understand more about your child's abilities 3 or more years after their Selective Dorsal Rhizotomy (SDR) operation.

Who is answering the survey?

- ☐ **Mother**
- ☐ **Father**
- ☐ **Both parents**
- ☐ **Other (Please specify………………………………………………………….)**

Tell us about your child

1. **How old was your child on their last birthday?**

   Age in years ………………..

2. **How old was your child when they had SDR surgery at Great Ormond Street Hospital?**

   Age in years …………………

3. **Is your child currently attending….**

   - ☐ Mainstream school – **No** Special Education Need (SEN) support or Education and Health Care Plan (EHCP)
   - ☐ Mainstream school – **With** Special Education Need (SEN) support or Education and Health Care Plan (EHCP)
   - ☐ Special Needs School
   - ☐ Home school
   - ☐ College
   - ☐ University
   - ☐ Apprenticeship
   - ☐ Work/ Employment
   - ☐ Other (Please specify……………………………………………………………………………)

4. **Does your child have any of the following conditions (other than cerebral palsy)? Please tick all that apply.**

☐ Epilepsy

☐ Autism Spectrum Disorder

☐ Attention Deficit Disorder (ADD) or Attention Deficit Hyperactivity Disorder (ADHD)

☐ Learning Disability

☐ Visual Impairment

☐ Hearing Impairment

☐ Physical condition other than cerebral palsy (please specify) ....

☐ Medical condition (please specify) ....

☐ Mental health needs (please specify) ....

☐ Under investigation/waiting for diagnosis (please specify)..

☐ Other (please specify) ....

☐ My child does not have any other condition

If your child does not have any other condition, go to question **6**

5. **Do you think any of the conditions selected above (if applicable) affect your child's day-to-day activities?**

	**Yes**	**No**	**Not Sure**
Epilepsy	☐	☐	☐
Autism Spectrum Disorder	☐	☐	☐
Attention Deficit Disorder (ADD) or Attention Deficit Hyperactivity Disorder (ADHD)	☐	☐	☐
Learning Disability	☐	☐	☐
Visual Impairment	☐	☐	☐
Hearing Impairment	☐	☐	☐
Physical condition other than cerebral palsy	☐	☐	☐
Medical condition (please specify)	☐	☐	☐
Medical condition	☐	☐	☐
Mental health needs	☐	☐	☐
Under investigation/waiting for diagnosis	☐	☐	☐
Other…	☐	☐	☐

Please specify how you think the selected condition(s) affect(s) your child's day-to-day activities.

We would like to know about medical or surgical treatments your child has needed SINCE the SDR surgery.

6. Has your child had oral medication or Botulinum toxin injections (Botox) for managing stiffness (spasticity/ dystonia) in your child's legs. Please tick all that apply.

	**Never** had this treatment for leg muscle stiffness	Needed this treatment **before SDR** but not since SDR	Needed this treatment **after SDR** but **not anymore**	**Still having** this treatment for leg muscle stiffness	**Cannot remember**
**Baclofen**	☐	☐	☐	☐	☐
**Trihexyphenidyl**	☐	☐	☐	☐	☐
**Gabapentin**	☐	☐	☐	☐	☐
**Botulinum Toxin injections (Botox)**	☐	☐	☐	☐	☐

If any other medication or any comments, please specify:

If 'Never had this treatment' or 'cannot remember', go to question **8**

7. **If your child had Botulinum toxin injections (Botox) for managing stiffness in their leg muscles <u>SINCE</u> the SDR surgery, please select which muscles were injected and how often <u>AFTER</u> SDR**

	How many times **in total**?
**Hamstrings**<br>(Muscles at the back of the thigh/knee)	
**Hip Adductors**<br>(Muscles on the inner side of the thigh, which pull legs close together)	
**Calf muscles**<br>(Muscles on the back of the lower leg)	

**Please specify if any other muscle group or any other comments about the 'botox' injections.**

8. **<u>Since</u> the SDR surgery, has your child needed orthopaedic surgery?**

- ☐ No surgery since SDR
- ☐ Yes, 1 operation
- ☐ Yes - 2 operations
- ☐ Yes- 3 operations
- ☐ Yes – more than 3 operations
- ☐ They are on the waiting list for surgery

If '**Yes**', go to question **9**

If '**waiting for surgery'**, go to question **10**

If '**No surgery since SDR'**, go to question **<u>11</u>**

9. **If yes, please select which surgery, how many times and when? Leave it blank if it doesn't apply.**

	How many times **in total?**	When did your child have the **first** orthopaedic surgery after SDR?	When did they have the **second** orthopaedic surgery after SDR? (if applicable)
**Calf muscles/ heel cords** (back of the lower leg			
**Hamstrings muscles** (muscles at the back of the thigh/knee)			
**Hip Adductors muscles** (muscles on the inner side of the thigh, which pull legs close together)			
Ankle/ Foot bony surgery			
Knee – bony surgery			
Hip- bony surgery			
Spinal surgery			

10. If waiting for surgery, please select which surgery.

- ☐ Calf muscles/ heel cords (back of the lower leg)
- ☐ Hamstrings (muscles at the back of the thigh/knee)
- ☐ Hip adductors (muscles on the inner side of the thigh, which pull legs together)
- ☐ Ankle/Foot – bone surgery
- ☐ Knee- bone surgery
- ☐ Hip- bone surgery
- ☐ Other (please specify): ………………………………………………………………………………………….

Any additional information on orthopaedic surgery such as procedures, dates:

We would like to know more about your expectations before SDR surgery and how you feel about SDR now.

11. Thinking back, what were your MAIN goals for your child undergoing SDR? (Please select your TOP 5 goals and rank these with 1 being the topmost goal)

	GOAL	RANK your top 5 goals only, with one being most important	Optional additional comments about the selected goal(s)
☐	to walk with ease		
☐	to reduce leg pain		
☐	to increase independence (in daily life skills, such as dressing, toileting)		
☐	to reduce leg stiffness		
☐	to improve posture		
☐	to be more comfortable generally		
☐	to walk longer distances		
☐	to keep up with peers		
☐	to reduce fatigue (tiredness)		
☐	to avoid repeated botulinum toxin injections		
☐	to avoid (minimise) future orthopaedic surgeries		
☐	to participate or improve participation in physical activities or sports		
☐	to participate or improve participation in recreational, leisure activities dance/ drama		
☐	Other (please specify) ...		
☐	Cannot remember		

**Comments:**

12. Have your expectations of SDR been met for each of your main 5 goals above?

	Mostly	Somewhat	Very little	Not at all	I am not sure
Goal 1	☐	☐	☐	☐	☐
Goal 2	☐	☐	☐	☐	☐
Goal 3	☐	☐	☐	☐	☐
Goal 4	☐	☐	☐	☐	☐
Goal 5	☐	☐	☐	☐	☐

Additional comments about why you think goals have been met or not:

13. How satisfied are you with your decision that you made for your child to undergo SDR?

☐ Very satisfied
☐ Satisfied
☐ Unsatisfied
☐ Very unsatisfied
☐ Neither satisfied/ nor unsatisfied

Please give reasons for your choice above:

14. Would you recommend the SDR procedure to other families with children who are similar in presentation to your child?

☐ Yes
☐ No
☐ Not Sure

Please give reasons for your choice:

We would like to know more about the longer-term effects of SDR

15. Does your child experience any unusual feelings (sensitivity) in their legs?

☐ Yes
☐ No
☐ Not sure

16. If yes or not sure, please describe what type of altered sensation (e.g. numbness, pins and needles, no feeling, ticklish etc.) do they experience and if this affects their daily activities?

17. Have you been told that your child has SCOLIOSIS of the spine? Scoliosis means that the spine is not straight but curves to the side, and it is diagnosed by X-ray and clinical examination. It is different from just a lean, which children can correct.

☐ Yes
☐ No
☐ Not sure

18. If yes, how is the scoliosis managed? Please select all that apply.

☐ Reviewed by physiotherapist
☐ Reviewed by orthopaedic/ spinal consultant
☐ Wears spinal brace
☐ Had spinal surgery
☐ On waiting list for spinal surgery
☐ Not being reviewed
☐ Other: (please specify)……………………………………………….

19. Has your child ever had any problems with bladder or bowel function?

	Never had this problem	This problem is new and started after SDR	Had this problem before SDR but it is better now	The problem has stayed the same before and after SDR	Had this problem before SDR but it is worse now	Not sure
Urinary incontinence (involuntary leakage of urine)	☐	☐	☐	☐	☐	☐
Increased urgency to pass urine	☐	☐	☐	☐	☐	☐
Constipation	☐	☐	☐	☐	☐	☐
Bowel incontinence (poo leakage)	☐	☐	☐	☐	☐	☐

20. Please indicate your level of agreement with the following statements. Since the SDR surgery, my child...

	**Strongly Agree**	**Agree**	**Neither Agree/ Disagree**	**Disagree**	**Strongly Disagree**	**Not applicable (did not have any concern before SDR)**	**Not sure if related to SDR**
Sleeps better	☐	☐	☐	☐	☐	☐	☐
Has less pain in their legs	☐	☐	☐	☐	☐	☐	☐
Feels less tired	☐	☐	☐	☐	☐	☐	☐
Is more comfortable in sitting	☐	☐	☐	☐	☐	☐	☐
Finds it easier to stand	☐	☐	☐	☐	☐	☐	☐
Finds it easier to walk	☐	☐	☐	☐	☐	☐	☐
Finds it easier to run	☐	☐	☐	☐	☐	☐	☐
Can participate better in activities with friends/ family	☐	☐	☐	☐	☐	☐	☐
Can participate better in physical activities/sports/ recreational activities	☐	☐	☐	☐	☐	☐	☐
Can do more things they like	☐	☐	☐	☐	☐	☐	☐
Is generally happier	☐	☐	☐	☐	☐	☐	☐
**Other (please specify)**							

21. Please indicate your level of agreement with the following statements in relation to your child's ability to participate in school/ college/university. Since the SDR surgery, my child...

	**Strongly Agree**	**Agree**	**Neither Agree/ Disagree**	**Disagree**	**Strongly Disagree**	**Not applicable (did not have any concern before SDR)**	**Not Sure if related to SDR**
Is more comfortable in sitting during lessons, group work	☐	☐	☐	☐	☐	☐	☐
Can move around the classroom easily	☐	☐	☐	☐	☐	☐	☐
Can move around the school/ college/ university easily	☐	☐	☐	☐	☐	☐	☐
Can participate better in physical activities with their friends (For example, physical education sessions)	☐	☐	☐	☐	☐	☐	☐
Can participate better in school events (For example, school trips, sports day, drama)	☐	☐	☐	☐	☐	☐	☐
Other (please specify)							

22. Have there been any other important changes (good or not) that you thought were related to the SDR surgery? (For example, the ability to use their hands, clarity in speech, behaviour, squint, cognition, attention)

☐ Yes
☐ No
☐ Not Sure

**If yes or not sure, please specify…**

We are interested in understanding your views on the need for the psychological (emotional) support before and after SDR.

23. From your own experience of your child undergoing SDR, do you feel YOU needed or would have benefited from psychological support *(For example, speaking with a psychologist or a family support worker)***?**

	Yes	No	Not Sure
Before SDR	☐	☐	☐
Immediately after SDR (during the hospital stay)	☐	☐	☐
First 6 months after SDR	☐	☐	☐
From 6 months to 2 years after SDR	☐	☐	☐
More than 2 years after SDR	☐	☐	☐

24. Do you feel YOUR CHILD needed or would have benefited from psychological support *(For example, speaking with a psychologist or a family support worker)***?**

	Yes	No	Not Sure
Before SDR	☐	☐	☐
Immediately after SDR (during the hospital stay)	☐	☐	☐
First 6 months after SDR	☐	☐	☐
From 6 months to 2 years after SDR	☐	☐	☐
More than 2 years after SDR	☐	☐	☐

Comments:

Please tell us about your child's current mobility and function

25. Does your child use any walking aids?

☐ Yes
☐ No- struggles to walk with walking aids
☐ No- does not need walking aid any more
☐ No- does not want to use walking aid
☐ No- never used any walking aid

26. Does your child use any mobility aids (wheelchair)?

- ☐ Yes
- ☐ No- does not need mobility aids any more
- ☐ No- does not want to use mobility aids
- ☐ No- never used a mobility aid

27. If YES, which walking mobility aid do they currently use and how often? Select all that apply.

	Always	Often	Sometimes	Rarely
Supportive walker (with seat and/or chest support)	☐	☐	☐	☐
Posterior walker (K-walker)	☐	☐	☐	☐
Anterior walker (walker in front)	☐	☐	☐	☐
Tripod/ Quad sticks	☐	☐	☐	☐
Elbow crutch sticks	☐	☐	☐	☐
Single point sticks (canes)	☐	☐	☐	☐
Wheelchair- self-propelled	☐	☐	☐	☐
Wheelchair- attendant propelled	☐	☐	☐	☐
Wheelchair - electric	☐	☐	☐	☐
**If using any other walking or mobility aids, or if you have any comments, please specify.**				

28. Does your child use any ankle foot orthotics (Splints/ insoles/ adaptive shoes etc.)?

- ☐ Yes
- ☐ No- does not tolerate orthotics
- ☐ No- does not want to wear orthotics
- ☐ No- does not need orthotics now
- ☐ No- never used any orthotics

29. If YES, which orthotics do they currently use and how often? Select ALL that apply

	**Uses all the time**	**Uses most of the time**	**Uses sometimes**	**Uses rarely**
Fixed ankle foot Orthotics (AFOs)	☐	☐	☐	☐
Hinged Ankle foot orthotics (flexible moving ankle joint)	☐	☐	☐	☐
Carbon fibre orthotics	☐	☐	☐	☐
A small splint that comes up to the ankle joint (Dynamic Ankle Foot orthosis /Supra Malleolar Orthoses)	☐	☐	☐	☐
Specialist supportive boots/footwear	☐	☐	☐	☐
Insoles/ heel cups	☐	☐	☐	☐
Night-time orthotics only	☐	☐	☐	☐

**If using any other orthotics or if have any comments, please specify:**

We would like to know about your child's current physiotherapy input This could be a single review, a block of treatment over a few weeks, days, months, or regular sessions

30. Does your child receive regular physiotherapy?

☐ Yes, regular sessions
☐ Yes, physiotherapy blocks
☐ Yes, reviews only
☐ No- my child does not receive physiotherapy
☐ Other: ……………………………………………………..

31. Thinking back in <u>the last 3 months,</u> approximately how often has your child received physiotherapy input?

**Physiotherapy input provided by:**	**How often?**					
	More than once a week	Once a week	Once every 2 weeks	Once a month	Once every 2 months	Once every 3 months
NHS/Local Health care physio	☐	☐	☐	☐	☐	☐
Self-funded/ Private physio	☐	☐	☐	☐	☐	☐
School exercise programme, supported by teaching/ physiotherapy assistant.	☐	☐	☐	☐	☐	☐

32. **Thinking back in <u>the last 6 months,</u> approximately how often has your child received a block of physiotherapy sessions?**

	**How many blocks?**	**How many days per week?**	**How many weeks?**
**NHS/ Local healthcare**			
**Self-funded/ Private physiotherapy**			
**Other**			

33. **On average, how many days per week does your child participate in physical activities such as swimming, bike/ trike, (frame) football, dance, martial arts, (frame) running, long walks, wheelchair sports or other similar activities?**

Days per week ……………………………

34. **If your child does not participate in physical activities, what are the reasons?**

- ☐ My child does not enjoy physical activities
- ☐ My child does not have easy access to physical activities
- ☐ My child cannot join in physical activities because of their physical difficulties in standing, walking or sitting
- ☐ My child cannot join in the activities because of their other needs (other than physical difficulties)
- ☐ Other reasons (please specify): ……………………………………………………………………….

35. **Is there anything else you would like to share with us about your experience of your child undergoing SDR?**

**Comments:**

36. Would you, as part of this study, be interested in an assessment of your child's abilities now, similar to previous clinical follow-up assessments before and after SDR?

- ☐ Yes
- ☐ No
- ☐ Would like more information

37. Would you, as part of this study, be interested in sharing your experiences with us through an in-person or a virtual interview?

- ☐ Yes
- ☐ No
- ☐ Would like more information

Thank you for taking the time to complete the questionnaire.
