# Supplementary material for: Mixed-methods study exploring medium to longer-term outcomes following selective dorsal rhizotomy in ambulatory children with cerebral palsy at a tertiary hospital in the UK: MOSAiC study protocol
Source: BMJ Open. 2025 Dec 8;15(12):e108558. doi: 10.1136/bmjopen-2025-108558 (PMC12699567; doi:10.1136/bmjopen-2025-108558)
Supplement: online supplemental file 4 [file bmjopen-15-12-s004.pdf]

Supplementary Information-4

INTERVIEW INDICATIVE TOPIC GUIDES

Parents/ Carers

This is an indicative topic guide for semi-structured interviews with parents/carers. Questions may not be asked in the order presented, nor phrased exactly as shown.

Topics to be covered	Questions	Prompts/Considerations
**Warm up question**	Could you just tell me a little bit about your child?	
**Why SDR? (decision-making)**	Could you describe why you decided your child should have SDR?<br><br>"CYP" was "x years old" at the time of surgery, what do you think was his/her understanding?	• How did SDR as a treatment option first come up?
**Parental Expectations**	What were your expectations of SDR for your child?	• What did you think this would look like for your child?<br>• What did you think short term/long term?
**CYP's expectation** (depending upon age at surgery)	Did they (child) have any goals/ expectations of their own?	
**Outcomes**	Were the outcomes / results of the surgery as expected/hoped?	• What was good, if anything?<br>• What was not so good?
**Emotional /psychological impact**	Was there any impact on you over the years after SDR?<br><br>Was there any impact on your child over the years after SDR?	• How prepared were you?<br>• How prepared was your child?
	Which of these outcomes /changes you have mentioned have had the most significant impact on your child's after SDR?<br><br>…your (families') life?	• How relevant/ meaningful are these changes to you?<br>• ...to your child?
**Other Factors**<br><br>Social Environmental and personal	Have there been any other factors that have been helpful or challenging for you and 'CYP' over the years?	This could be related to anything…such as family, school, other treatments/surgeries, equipment, physiotherapy, or other activities

**Parental Experience**	How would you describe your overall experience after two years and until now?	• What was helpful? What was challenging?
	Has your child undergone any other interventions since SDR?	• Was this something you were expecting, or not?<br>• How did you / your child feel about it?
**Perception of SDR now**	Having gone through the process XX years later how do you feel about SDR now?	
	What do you hope for your child in the future?	
	What advice would you give to a family about to undertake the process of SDR?	
**Closing question**	Anything further you would like to add to already what has been discussed.	

Interview Indicative Topic Guide: Children and Young People

The interview topic guide will be adapted for CYP depending upon the child's age at interview, understanding and age at surgery. CYP will be given a choice on what they would like to talk about first (Friends, Family, Fun, Fitness, Functioning and Future).

Topics to be covered	Questions	Prompts/Considerations
**Warm up Question**	Tell me what you like doing during your day?	
**Participation (Fun, Friends, Family)**	What activities do you like to do?	• Is there anything that is not so much fun?<br>• How do you feel about it?<br>• Is there anything that's tricky or perhaps very easy?
**Functioning (School/college)**	Tell me about your ...School / College<br><br>How do you feel things are going at school?	• What do you like doing?<br>• What is not so much fun?<br>• What's tricky?
**Functioning (Home/ community)**	How do you feel about all the activities that you have to do at home?	• Is there anything stopping you from doing what you want to do or would like to do?<br>• What do you think would help you to do what you want to do?
**About SDR**	Can you tell me what you remember about your operation (SDR)?<br><br>Can you remember what you were like before the operation?	• What was it like for you?<br>• What was good?<br>• What was not so good?<br><br>What was tricky for you?
**Goals of SDR**	Do you know why you had this surgery?	• Was there anything in particular that you wanted to be to be able to do after SDR?
**Post-SDR rehabilitation (Fitness)**	How do you feel about the physiotherapy/ exercises that you had to do after SDR?<br><br>Do you do physiotherapy/ exercises now?	• How do you feel about it?

**Outcomes after SDR**	How do your body/legs/ muscles feel now?	• How do you feel in the morning/ at the end of the school/college day?<br><br>(Muscle tightness/ weakness/ Sensation/ Fatigue/ pain)
**Future Goals and Aspirations**	What would you like to be able to do in future?	• It could be next week/ month/ year?
**Advice to others**	What would you tell another child who is thinking of the SDR operation?<br><br>Do you have a message for parents/ health professionals about SDR?	
**Closing question**	Is there anything else you would like to tell me, that you think is important?	
