# Supplementary material for: Mixed-methods study exploring medium to longer-term outcomes following selective dorsal rhizotomy in ambulatory children with cerebral palsy at a tertiary hospital in the UK: MOSAiC study protocol
Source: BMJ Open. 2025 Dec 8;15(12):e108558. doi: 10.1136/bmjopen-2025-108558 (PMC12699567; doi:10.1136/bmjopen-2025-108558)
Supplement: Supplementary file 5 [file bmjopen-2025-108558.reviewer_comments.pdf]

PEER REVIEW HISTORY

BMJ Open publishes all reviews undertaken for accepted manuscripts. Reviewers are asked to complete a checklist review form and are provided with free text boxes to elaborate on their assessment. These free text comments are reproduced below.

ARTICLE DETAILS

Title (Provisional)

MOSAiC study protocol: A mixed methods study exploring Medium to longer term Outcomes following Selective dorsal rhizotomy in Ambulatory children with Cerebral palsy at a tertiary hospital in the UK

Authors

Chugh, Deepti; Kilbride, Cherry; Gimeno, Hortensia; Aquilina, Kristian; Alderson, Lucy; Theologis, Tim; Main, Eleanor

---

This article was not externally reviewed at BMJ Open. Protocols that have been independently assessed prior to submission to BMJ Open are usually fast-tracked to publication on the grounds that further substantial changes will not be possible. This independent assessment will usually be external, independent review for both a substantial grant award from a non-commercial or government funder and ethics approval.
